# Weighing options: empiric antibiotic use and stewardship opportunities in critically ill patients with community-acquired pneumonia

**DOI:** 10.1017/ash.2025.10082

**Published:** 2025-08-07

**Authors:** Nalea Trujillo, Calvin Diep, David Ha, Ariadna Garcia, Marisa Holubar

**Affiliations:** 1 Department of Pharmacy, Stanford Health Care, Stanford, CA, USA; 2 Stanford University School of Medicine, Quantitative Sciences Unit, Stanford, CA, USA; 3 Division of Infectious Diseases and Geographic Medicine, Stanford University School of Medicine, Stanford, CA, USA; 4 Department of Quality, Stanford Health Care, Stanford, CA, USA

## Abstract

In this retrospective study, critically ill patients with community-acquired pneumonia frequently received empiric anti-methicillin-resistant Staphylococcus aureus (MRSA) and antipseudomonal antibiotics despite having few or no guidelines-endorsed risk factors. De-escalation of anti-MRSA therapy was quicker, likely aided by MRSA polymerase chain reaction assays.

## Introduction

Community-acquired pneumonia (CAP) is associated with high morbidity and mortality.^1^ Up to 40% of hospitalized patients with CAP receive empiric broad spectrum antibiotics against methicillin-resistant *Staphylococcus aureus* (MRSA) or *Pseudomonas aeruginosa* despite low prevalence of these pathogens (<4%).^2–5^ In the intensive care unit (ICU) frequent empiric broad spectrum antibiotics and delayed or limited de-escalation are common often due to severity of illness and patient complexity,^6^ making ICU CAP an important target for antibiotic stewardship.

Current IDSA/ATS guidelines for hospitalized patients with CAP recommend empiric anti-MRSA or antipseudomonal antibiotics in patients with prior respiratory isolation of these organisms within the last year or in those hospitalized with receipt of parenteral antibiotics within the last 90 days.^1^ We sought to provide insight into the dynamics of antibiotic management in ICU patients with CAP as they relate to patient factors and microbiological testing, with particular focus on broad spectrum anti-MRSA and antipseudomonal agents.

## Methods

This was a retrospective, single-center study at Stanford Health Care, a 613-bed academic medical center. Patients were included if they were > 18 years old, admitted to an ICU between January 1, 2022 and December 31, 2023, and received systemic antibiotic therapy for pneumonia within the first 24 hours of admission. Patients were excluded if they had a history of lung transplant, bone marrow transplant, cystic fibrosis, had suspected extrapulmonary infection, lung abscess, empyema, severe neutropenia (ANC <500 cells/mL), required chronic mechanical ventilation prior to admission, were transferred from an outside hospital, or were discharged or transitioned to comfort care within 48 hours of initiating antibiotics. “Guidelines-endorsed risk factors” for MRSA and *P. aeruginosa* were defined in accordance with IDSA/ATS CAP guidelines. Data was collected by review of the electronic medical record or extracted from an institutional research database. This study was approved by the Stanford University Institutional Review Board.

### Data analysis

Categorical variables were analyzed using either Pearson’s Chi-squared test or Fisher’s exact test. For continuous variables, the Wilcoxon rank sum test was applied. To identify factors associated with empiric anti-MRSA and antipseudomonal therapy, adjusted mixed-effects multivariate logistic regression models were utilized. The odds ratios (OR) and corresponding 95% confidence intervals (CIs) were reported for each type of therapy. Additionally, we examined the proportion of patients de-escalated from therapy within a 7-day period, stratified by risk factors, polymerase chain reaction (PCR) results, and the type of therapy administered. We also described the proportion of patients who received prolonged therapy (> 3 d).

## Results

We screened 435 patients and 169 were included in the final analysis (Supplemental Figure 1). Baseline characteristics are shown in supplemental Table 1. Of those included, 57% (97/169) of patients required mechanical ventilation and/or vasopressors and 10% (17/169) and 11% (18/169) had at least 1 guidelines-endorsed risk factors for MRSA and *P. aeruginosa*, respectively. When a respiratory culture was obtained 4% (3/82) and 5% (4/82) grew MRSA and *P. aeruginosa* respectively (Supplemental Table 2). Regarding empiric antibiotic prescribing, 47% (79/169) received anti-MRSA therapy and 62% (105/169) received antipseudomonal therapy.

**Figure 1.**
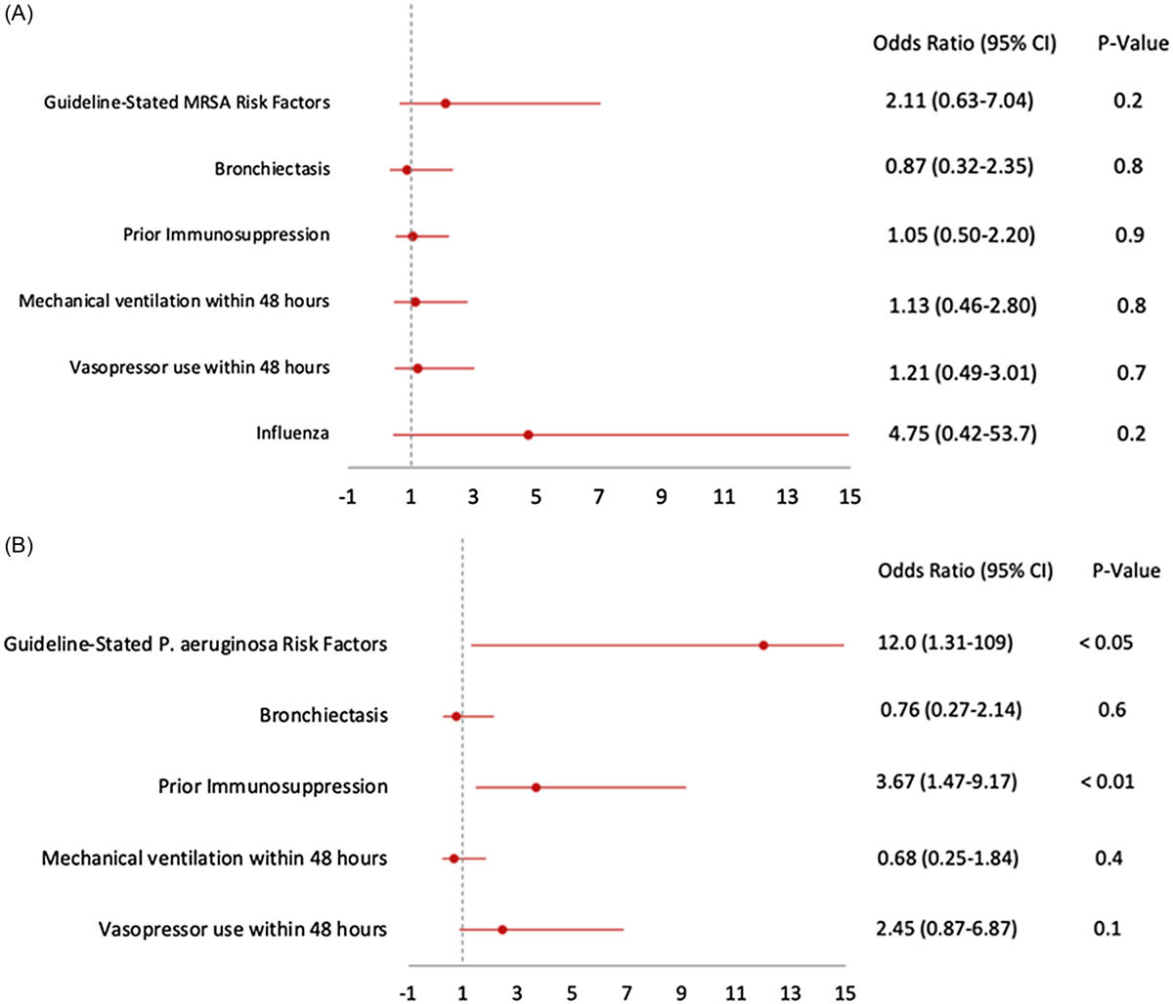
A. Mixed-effects logistical regression for factors associated with empiric anti-MRSA therapy. B. Mixed-effects logistical regression for factors associated with empiric antipseudomonal therapy.

**Figure 2.**
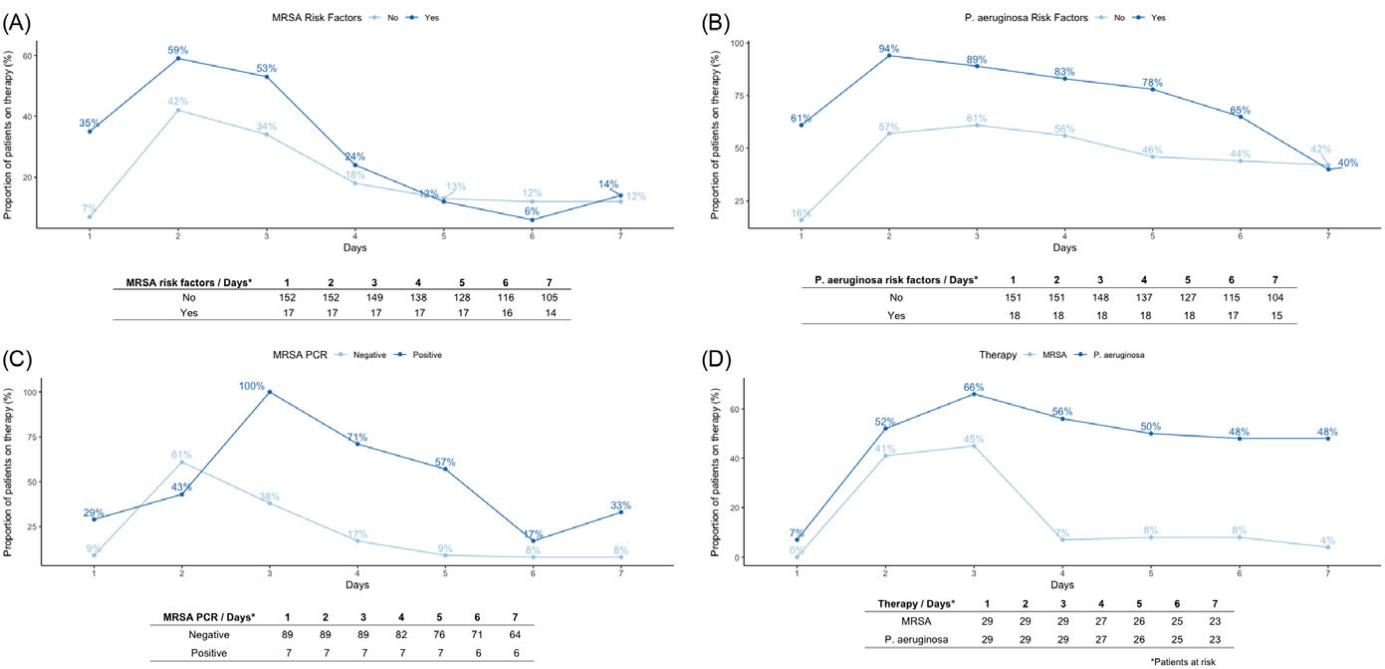
A. Proportion admitted receiving anti-MRSA therapy based on presence of MRSA risk factors. B. Proportion admitted receiving antipseudomonal therapy based on presence of pseudomonas risk factors. C. Proportion admitted receiving anti-MRSA therapy based on MRSA PCR results. D. Proportion receiving anti-MRSA and antipseudomonal therapy in patients with normal flora on respiratory cultures.

Guidelines-endorsed risk factors for *P. aeruginosa* and immunosuppression were associated with empiric antipseudomonal therapy while no factors were associated with empiric anti-MRSA therapy (Figure 1). Of those who received empiric anti-MRSA or antipseudomonal therapy, 14% (11/169) and 16% (17/169) had risk factors, respectively (Supplemental Tables 3 and 4). De-escalation of anti-MRSA therapy was performed earlier and more often than antipseudomonal therapy. In patients with negative PCR nasal screening 17% were on anti-MRSA therapy at day 4 versus 71% of patients with a positive result. In patients with only normal flora isolated from respiratory cultures, anti-MRSA therapy was de-escalated within 3–4 days of admission, while antipseudomonal therapy was rarely de-escalated (Figure 2). Prolonged (>3 d) anti-MRSA therapy was more likely in patients with a positive nasal MRSA PCR 44% vs 3.4%; *P* = .001 despite negative respiratory and blood cultures (Supplemental Table 5). Prolonged antipseudomonal therapy was more likely in patients with *P. aeruginosa* risk factors 17% vs 4.5%; *P* = .009, severe CAP 64% vs 42%; *P* = .006, mechanical ventilation 55% vs 32%; *P* = .002, and vasopressor use 53% vs 37%; *P* = .006 (Supplemental Table 6).

## Discussion

In this single-center study of antibiotic management of CAP in ICU patients, we found a high rate of empiric anti-MRSA and antipseudomonal antibiotic use despite low prevalence of guidelines-endorsed risk factors and low rate of recovery of these organisms from culture. ICU admission has been associated with greater broad spectrum antibiotic use, possibly due to increased severity of illness and patient complexity.^4–5^ These data suggest that there is antibiotic stewardship opportunity to align empiric antibiotic prescribing with those at greatest risk of drug-resistant organisms.

In our study, drivers of empiric antipseudomonal therapy included presence of guidelines-endorsed risk factors and immunosuppression, while no clear drivers of anti-MRSA therapy were identified. Jones et al found that patients receiving empiric anti-MRSA therapy had greater comorbidity burden (ie, renal disease, neoplastic disease, nursing home resident), more risk factors for MRSA, and greater severity of illness.^5^ Angrill et al found that prior hospitalization, severe COPD, and immunosuppression were associated with antipseudomonal therapy.^6^ Current guidelines for treatment of cancer-related infections recommend empiric coverage for *P. aeruginosa,* which likely explains association between immunosuppression and antipseudomonal agent use seen in this study as well as Angrill et al.^7^ The differences in institutional practice, local resistance patterns and patient population may explain why risk factors for empiric anti-MRSA and antipseudomonal therapy varied across these studies.

We found frequent de-escalation of anti-MRSA therapy in response to negative MRSA PCR results. We previously reported this and now demonstrate this intervention’s durability.^8^ In contrast, we did not identify any factors promoting antipseudomonal therapy de-escalation, which resulted in prolonged antibiotic durations. Severity of illness was a major driver of prolonged antipseudomonal therapy despite low recovery rates in culture. Negative respiratory cultures theoretically present an opportunity to de-escalate antipseudomonal therapy, however, our study, along with others, suggest limited clinician response to these results.^9,10^ Clinical response as well as worsening may invite the continuation of broad-spectrum antibiotics.

Our study has several limitations. The retrospective design limits understanding of CAP diagnosis and empiric prescribing and de-escalation decision-making. Approximately 50% of patients did not have a respiratory culture obtained, which may underestimate the culture-confirmed MRSA or *P. aeruginosa* cases. Respiratory cultures were more likely to be collected in patients with severe CAP (eg, requiring mechanical ventilation or vasopressors) (Supplemental Tables 3 and 4) so sampling was enriched for higher risk patients. The absence of respiratory cultures is reflective of real-world practice as patients may not produce adequate sputum and the use of deep culturing techniques (bronchoalveolar lavage) are typically used in intubated patients. Finally, these results reflect local prescribing practices and epidemiology and may not be generalizable, however, similar prescribing trends have also been reported in other studies.^4,10^

These results demonstrate a low rate of MRSA and *P. aeruginosa* CAP in critically ill patients and outline opportunities to improve empiric broad spectrum antibiotic prescribing and de-escalation. Assessing incidence of and risk factors for MRSA and *P. aeruginosa* presents a means to reduce empiric broad spectrum antibiotic use while the use of MRSA PCR testing and respiratory cultures can promote de-escalation. We posit that our methods may be employed by others to reveal institution-specific opportunity in this often challenging critically ill population.

## Supporting information

10.1017/ash.2025.10082.sm001 Trujillo et al. supplementary material Trujillo et al. supplementary material

## Supplementary material

The supplementary material for this article can be found at https://doi.org/10.1017/ash.2025.10082.
