# Supplementary material for: Weighing options: empiric antibiotic use and stewardship opportunities in critically ill patients with community-acquired pneumonia
Source: Antimicrob Steward Healthc Epidemiol. 2025 Aug 7;5(1):e180. doi: 10.1017/ash.2025.10082 (PMC12345047; doi:10.1017/ash.2025.10082)
Supplement: Trujillo et al. supplementary material [file S2732494X2510082Xsup001.docx]

**Supplemental Material**

Table of Contents

[Supplemental Table 1: Baseline Characteristics 2](#_Toc200997468)

[Supplemental Table 2: Respiratory Culture Data 3](#_Toc200997469)

[Supplemental table 3: Culture data for patients on mechanical ventilation 4](#_Toc200997470)

[Supplemental table 4: Culture data for patients on vasopressors 4](#_Toc200997471)

[Supplemental Table 5: Concordance with Guideline-State Risk Factors vs Empiric Anti-MRSA Therapy 4](#_Toc200997472)

[Supplemental Table 6: Concordance with Guideline-Stated Risk Factors vs Empiric Antipseudomonal Therapy 4](#_Toc200997473)

[Supplemental Table 7: Prolonged Anti-MRSA Therapy (>3 days) 5](#_Toc200997474)

[Supplemental Table 8: Prolonged Antipseudomonal Therapy (>3 days) 5](#_Toc200997475)

**Definitions**

| **Outcome** | **Definition** |
| --- | --- |
| Empiric anti-MRSA therapy | Patients who received anti-MRSA therapy within the first 48 hours of admission |
| Empiric anti-PSA therapy | Patients who received antipseudomonal therapy within the first 48 hours of admission |
| Prolonged therapy | Empiric anti-MRSA or antipseudomonal therapy > 3 days. Greater than 3 days was chosen as a pragmatic timepoint as cultures would have generally resulted by this time providing an opportunity to de-escalate or continue anti-MRSA or anti-pseudomonal therapy. |
| Therapy de-escalation | Receipt of anti-MRSA or antipseudomonal therapy for ≥ 1 day followed by ≥ 2 days without anti-MRSA or antipseudomonal therapy |

**Supplemental Figure 1: Patient Consort Diagram**


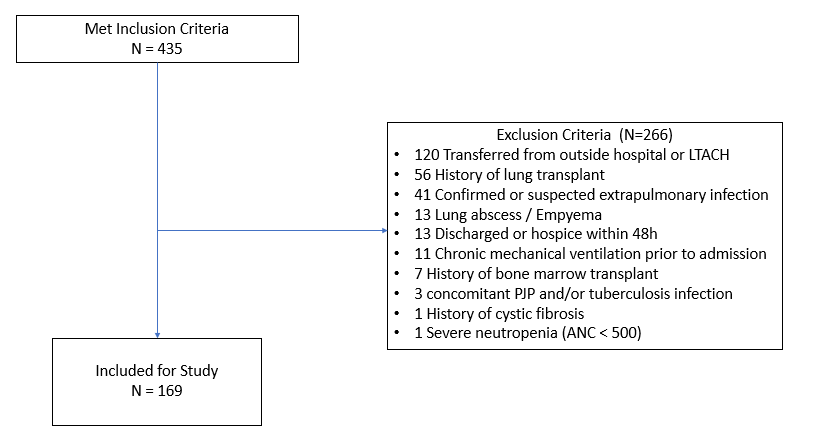


# Supplemental Table 1: Baseline Characteristics

| Variable | N=169 |
| --- | --- |
| Age (years), median (IQR) | 71 (53, 83) |
| Male, n (%) | 90 (53) |
| Comorbidities, n (%)  Bronchiectasis  Chronic kidney disease  Dialysis dependent  Type 2 diabetes mellitus  Chronic obstructive pulmonary disease  Human immunodeficiency virus  Solid organ malignancy | 24 (14)  56 (33)  17 (10)  73 (43)  39 (23)  1 (1)  66 (39) |
| Solid organ transplant, n (%)  Any  Heart  Kidney  Liver | 7 (4.1)  1 (1)  4 (2.4)  3 (1.8) |
| Concomitant respiratory viral infection, n (%)  Influenza  COVID-19  Other respiratory virus | 5 (3)  19 (11)  13 (8) |
| MRSA and *P. aeruginosa* risk factors  MRSA in respiratory culture in past 1 year  *P. aeruginosa* in respiratory culture in past 1 year  Hospitalization and intravenous antibiotic use in past 90 days | 1 (1)  1 (1)  17 (10) |
| Severe community-acquired pneumonia (Major determinants), n (%)  Any  Mechanical ventilation within 48 hours  Vasopressor use within 48 hours | 97 (57)  73 (4)  75 (45) |
| Severe community-acquired pneumonia (Minor determinants), n (%)  Three or more minor determinants  Tachypnea  Confusion/disorientation  Uremia  Leukopenia  Thrombocytopenia  Hypothermia | 23 (14)  34 (20)  28 (17)  86 (51)  11 (7)  10 (6)  97 (57) |
| Prior admission immunosuppression, n (%)  Any  Chemotherapy  High-dose corticosteroids  Calcineurin inhibitor or antiproliferative agent  AIDS-defining conditions  Leukemia  Lymphoma  Solid organ transplant  CD4 < 200 | 51 (30)  26 (15)  9 (5.3)  12 (7)  1 (1)  5 (3)  3 (2)  7 (4)  1 (1) |
| Pneumonia on imaging, n (%)  Chest X-ray and CT  Chest X-ray  Chest CT  None | 88 (52)  66 (39)  4 (2.4)  11 (7) |
| Enteral tube feeds | 85 (51) |
| Presence of central access | 73 (43) |
| ICU length of stay (days), median (IQR) | 6 (3, 10) |
| Hospital length of stay (days), median (IQR) | 10 (6, 17) |
| All-cause 30-day readmission, n (%) | 27 (16) |
| Acute-care exposure in past 90 days, n (%) | 66 (39) |

# Supplemental Table 2: Respiratory Culture Data

|  | N=169 |
| --- | --- |
| Culture Not Obtained, n (%) | 87 (51) |
| Culture Obtained^A^       Normal flora       Rejected by lab^B^       No growth       MRSA  *P. aeruginosa*  Other organisms^C^ | 82 (49)  41 (50)  22 (27)  6 (7)  3 (4)  4 (5)  6 (7) |
| Culture method^D^  Sputum  Tracheal aspirate  Bronchioalveolar lavage | 25 (42)  19 (32)  16 (27) |

^A^Percentages of culture results calculated based on denomentaor of 82 for culture obtained

^B^Typical reasons for rejection per lab protocol were (1) contamination noted by presence of excessive

squamous epithelial cells or (2) lack of positive gram stain for organisms or neutrophils on culture

specimen

^C^Other organisms: Corynebacterium striatum, methicillin-sensitive Staphylococcus aureus, Citrobacter freundii complex, non-fermenting GNR, lactose-fermenting GNR, Haemophilus influenzae, Aspergillus fumigatus, Escherichia coli, Klebsiella pneumoniae, Streptococcus dysgalactiae

^D^Percentages of culture method calculated based on denomenator of 60 for cultures obtained that were not rejected by lab

#

# Supplemental table 3: Culture data for patients on mechanical ventilation

|  | N = 73 |
| --- | --- |
| Culture Obtained^A^, n (%)  Normal flora  Rejected by lab  No growth  MRSA  *P. aeruginosa*  Other organisms^B^ | 43 (59)  23 (53)  6 (14)  6 (14)  1 (2)  3 (7)  4 (9) |

^A^Percentages of culture results calculated based on denomentaor of 43 for culture obtained

^B^MSSA

# Supplemental table 4: Culture data for patients on vasopressors

|  | N = 75 |
| --- | --- |
| Culture Obtained^A^       Normal flora       Rejected by lab       No growth       MRSA  *P. aeruginosa*  Other organisms^B^ | 42 (56)  19 (45)  8 (19)  7 (17)  2 (5)  2 (5)  4 (10) |

^A^Percentages of culture results calculated based on denomentaor of 42 for culture obtained

^B^MSSA, streptococcus dysgalactiae

# Supplemental Table 5: Concordance with Guideline-State Risk Factors vs Empiric Anti-MRSA Therapy

|  | Empiric Anti-MRSA Therapy | |
| --- | --- | --- |
|  | **Yes (N=79)** | **No (N=90)** |
| MRSA risk factors present | 11 (14%) | *6 (7%)* |
| MRSA risk factors absent | *68 (86%)* | 84 (93%) |

Bold and italics indicates guideline concordant and non-concordant prescribing, respectively.

# Supplemental Table 6: Concordance with Guideline-Stated Risk Factors vs Empiric Antipseudomonal Therapy

|  | Empiric Antipseudomonal Therapy | |
| --- | --- | --- |
|  | **Yes (N=105)** | **No (N=64)** |
| *P. aeruginosa* risk factors present | 17 (16%) | *1 (2%)* |
| *P. aeruginosa* risk factors absent | *88 (84%)* | 63 (98%) |

Bold and italics indicates guideline concordant and non-concordant prescribing, respectively.

# Supplemental Table 7: Prolonged Anti-MRSA Therapy (>3 days)

|  | Prolonged Anti-MRSA Therapy | | P-value |
| --- | --- | --- | --- |
|  | **No (N=150)** | **Yes (N=16)** |  |
| MRSA risk factors | 15 (10%) | 1 (6.3%) | >0.9 |
| MRSA PCR  Negative  Positive | 84 (97%)  3 (3.4%) | 5 (5.6%)  4 (44%) | 0.001 |
| Immunosuppresion | 48 (32%) | 3 (19%) | 0.4 |
| Influenza | 4 (2.7%) | 1 (6.3%) | 0.4 |
| Mechanical ventilation | 62 (42%) | 10 (63%) | 0.11 |
| Vasopressor use | 64 (43%) | 9 (56%) | 0.3 |
| Severe CAP | 76 (51%) | 11 (69%) | 0.2 |

# Supplemental Table 8: Prolonged Antipseudomonal Therapy (>3 days)

|  | Prolonged Anti-PsA Therapy | | P-value |
| --- | --- | --- | --- |
|  | **No (N=88)** | **Yes (N=77)** |  |
| *P. aeruginosa* risk factors | 4 (4.5%) | 13 (17%) | 0.009 |
| Respiratory cultures | 27 (31%) | 29 (38%) | 0.3 |
| Immunosuppression | 22 (25%) | 28 (36%) | 0.11 |
| Mechanical ventilation | 28 (32%) | 42 (55%) | 0.002 |
| Vasopressor use | 32 (37%) | 41 (53%) | 0.034 |
| Severe CAP | 37 (42%) | 49 (64%) | 0.006 |
| Bronchiectasis | 15 (17%) | 8 (10%) | 0.2 |
